# Identification of highly effective inhibitors against SARS-CoV-2 main protease: From virtual screening to in vitro study

**DOI:** 10.3389/fphar.2022.1036208

**Published:** 2022-11-18

**Authors:** Hu Wang, Jun Wen, Yang Yang, Hailin Liu, Song Wang, Xiaoli Ding, Chunqiao Zhou, Xuelin Zhang

**Affiliations:** ^1^ Department of Pharmacy, The First People’s Hospital of Chongqing Liang Jiang New Area, Chongqing, China; ^2^ Department of Pharmacology, Chongqing Health Center for Women and Children, Chongqing, China

**Keywords:** SARS CoV-2, main protease, virtual screening, molecular dynamics simulation, inhibitor

## Abstract

**Background and Objective:** The public’s safety has been significantly jeopardized by the pandemic of COVID-19, which is brought on by the highly virulent and contagious SARS-CoV-2 virus. Finding novel antiviral drugs is currently of utmost importance for the treatment of patients with COVID-19. Main protease (3CL^pro^) of SARS-CoV-2 is involved in replication of virus, so it is considered as a promising target. Using small molecules to inhibit SARS-CoV-2-3CL^pro^ activity may be an effective way to prevent viral replication to fight COVID-19. Despite the fact that some SARS-CoV-2-3CL^pro^ inhibitors have been described, only few of them have high levels of inhibition at nanomolar concentrations. In this study, we aimed to screen out effective SARS-CoV-2-3CL^pro^ inhibitors.

**Methods:** To identify highly effective SARS-CoV-2-3CL^pro^ inhibitors, a pharmacophore mapping and multiple-conformation docking were efficiently applied to find novel hit compounds from a database. Then, the stability of the 3CL^pro^-hit complexes was validated by using molecular dynamics simulation. Finally, biological assay was used to assess the inhibition effects of hit compounds on SARS-CoV-2-3CL^pro^.

**Results:** Four hit compounds were identified by using computer-assisted strategy. Molecular dynamics simulation suggested that these hits bound stably to the 3CL^pro^-active pocket. Bioassay showed that all the hits had potent inhibition against SARS-CoV-2-3CL^pro^ with IC_50_ values in the range of 0.017–0.83 μM. Particularly, hit one was the best 3CL^pro^ inhibitor and its inhibition effect of SARS-CoV-2-3CL^pro^ (IC_50_ = 0.017 ± 0.003 µM) was about 236 times stronger than that of ML300 (IC_50_ = 4.01 ± 0.66 µM).

**Conclusion:** These data indicate that hit one could be regarded as an anti-SARS-CoV-2 candidate worth exploring further for the treatment of COVID-19.

## 1 Introduction

Since December 2019, the pandemic of COVID-19 caused by SARS-CoV-2, has infected more than 166 million people and killed more than 3.5 million people worldwide (Koutsakos and Kedzierska, 2020; Nassau et al., 2022). SARS-CoV-2 usually induces respiratory symptoms, such as cough and fever (Guan et al., 2020; Narayanan et al., 2022). Although effective antiviral agents have been developed against COVID-19, the rapid mutation of the virus limits their clinical use. (Tao et al., 2021; Chakraborty et al., 2022). Some studies have shown that the mutations in the S-glycoprotein and RdRp of the genome cause therapeutic resistance of antibodies or small molecules (Tao et al., 2021; Chakraborty et al., 2022; Rockett et al., 2022; Stevens et al., 2022). Therefore, it is urgent to develop novel, highly potent antiviral candidates to treat COVID-19.

Efforts are under way to characterize molecular targets, which are essential for the development of anti-coronavirus drugs. SARS-CoV-2 main protease (3CL^pro^) is mainly involved in the cleavage of polyproteins (PPs) to generate non-structural proteins (NSPs), which are then compiled into replicase transcriptase complexes (RTC) (Jin et al., 2020). 3CL^pro^ is a dimer composed of two monomers that are arranged almost perpendicular to each other (Zehra et al., 2020). Each monomer has three distinct domains: Domains I and II display an antiparallel β-barrel structure and contain the catalytic dyad (His41 and Cys145), while domain III is a large antiparallel globular structure composed of five α-helices (Zehra et al., 2020). Because 3CL^pro^ of SARS-CoV-2 is involved in viral polyproteins processing and maturation (Xia and Kang, 2011), it is considered to be a promising target. 3CL^pro^ cleaves the initially translated viral polyproteins, generating nonstructural proteins, and then interferes with viral replication and maturation (Ferreira et al., 2021). Thus, inhibition of 3CL^pro^ activity would block viral replication (Vuong et al., 2020). In addition, it contains a unique recognition sequence Leu-Gln*Ser-Ala-Gly (* marks the cleavage site) during its cleavage function on viral polyprotein, which is unrecognized by any known human protease (Zehra et al., 2020). This suggests that compounds capable of inhibiting 3CL^pro^ are unlikely to be toxic (Adegbola et al., 2021). Therefore, SARS-CoV-2-3CL^pro^ is recognized as an effective drug target for treating COVID-19.

In view of the continuous variation of SARS-CoV-2, the clinical researches of some antibody drugs have stalled. However, the vast majority of molecules on 3CL^pro^ inhibitors have great potential in fighting against new coronavirus variants. The following are some advances in the development of drugs targeting SARS-CoV-2-3CL^pro^ (Supplementary Table S1). For example, paxlovir (PF-07321332), a first protease inhibitor against the SARS-CoV-2 protease 3CL^pro^, has recently been approved as an antiviral for SARS-CoV-2 by the United States Food and Drug Administration (FDA) (Abdelnabi et al., 2022). Tyndall et al. discovered that S-217622 is a noncovalent nonpeptide oral inhibitor against SARS-CoV-2-3CL^pro^ and the latest clinical results show that S-217722 has good antiviral activity and oral bioavailability (Tyndall, 2022). Boras et al. reported that PF-07304814 can act as a broad-spectrum coronavirus 3CL^pro^ inhibitor and showed potent antiviral activity *in vivo* (Boras et al., 2021). In addition, EDP-235 is a novel and highly selective 3CL^pro^ inhibitor and was evaluated in the first in-human phase I study in healthy volunteers for safety, tolerability, and pharmacokinetics (Hu et al., 2022).

Computer-aided drug design (CADD) including pharmacophore modeling and molecular docking has established itself as a valuable *in silico* technique for the identification of the probable inhibitors that could prevent the activity of an enzyme (Kitchen et al., 2004; Maga et al., 2008). Compared with the traditional drug discovery strategies, this method significantly decreases the time and cost to develop a new drug (Kitchen et al., 2004; Wang et al., 2005; Maga et al., 2008; Gao et al., 2010; Wieder et al., 2017). By the CACS strategy, researchers successfully identified a highly potent peptide, NKTP-3, with dual inhibitory effects on both NRP1 and KRAS^G12D^ (Zhou et al., 2022). Zheng et al. also discovered an anticancer agent TP-3 targeting tubulin and PARP-1 using a CACS approach (Zheng et al., 2021). Furthermore, Zhou et al. used a combination of pharmacophore and docking approaches to identify a first candidate peptide with dual-targeting both NRP1 and MDM2 (Zhou et al., 2021). These studies suggested that the combined screening is an attractive strategy in drug lead exploration.

In this study, with the recently resolved inhibitor-bound SARS-CoV-2-3CL^pro^ crystal structure available (Han et al., 2022), we constructed a structure-based pharmacophore model of 3CL^pro^. An efficient database screening strategy was used for the virtual screening of 3CL^pro^ inhibitors from a virtual database. Four hit compounds were finally identified based on low root-mean-square distance (RMSDx) values as well as better docking scores and subjected to molecular dynamics simulation. Biological assay showed that these four hit compounds had an inhibition effect on SARS-CoV-2-3CL^pro^ activity. These results indicate that the structure-based approach is also suitable for the discovery of SARS-CoV-2-3CL^pro^ inhibitors.

## 2 Materials and methods

### 2.1 Structure-based pharmacophore modeling

In this modeling study, we used the 3CL^pro^ (PDB code: 7LME) from SARS-CoV-2 in complex with ML300 as the crystal structure. We selected this crystal structure according to the following criteria (Tian et al., 2022): 1) The organism of the selected crystal structure should be SARS-CoV-2 rather than other species. The organism of the 3CLpro structure is SARS-CoV-2. 2) One of the quality indexes of protein crystal structure is resolution, which represents the uncertainty of atomic position in crystal structure model. When there are many crystal structures available, we choose the one with high resolution (that is, the one with small resolution value). Generally, structures with a resolution less than 3 Å are sufficient for pharmacophore modeling. The 3CL^pro^ crystal structure has a high resolution of 2.10 Å 3) The selected crystal structure should include an active pocket; the crystal structure of 3CL^pro^ contains the active-binding pocket. This structure file was prepared by the MOE program (Zhou et al., 2019a), with the following protocol: hydrogens were added, water molecules were removed, partial charges were computed and energy minimization was carried out using the Amber10:EHT forcefield. In the Pharmacophore Query Editor, PCH pharmacophore scheme including hydrogen-bond acceptor, aromatic, and hydrophobic feature was selected. Amber10:EHT forcefield was assigned to the system. Pharmacophore Query Editor was used to manually construct a visualized 3D-pharmacophore model by analyzing protein-ligand interaction in the 3CL^pro^ complex (Zhou et al., 2019a; Zhou et al., 2019b). In our study, the generated pharmacophore model was exported and translated into a pharmacophore file by a script.

### 2.2 Güner–Henry (GH) scoring method

According to a previously reported method (Zhou et al., 2019a), the GH method was used to evaluate precision of model selectivity. In the study, we used the generated pharmacophore model to successfully screen actives from a 1,090 molecular database. This database consists of the decoy set containing 1,080 molecules (retrieved from DUD-E database) (Mysinger et al., 2012) and 10 known 3CL^pro^ inhibitors (Han et al., 2022). The resulted mapping data was used to evaluate pharmacophore quality by solving the following equation (The GH score with greater than 0.6 indicated a good model):

$$GH=\left(\frac{Ha\left(3A+Ht\right)}{4HtA}\right)\left(1-\frac{\left(Ht-Ha\right)}{\left(D-A\right)}\right)$$

### 2.3 Virtual screening

The virtual screening was performed to screen a chemical database containing 35,000 molecules (Zhou et al., 2019a). In the course of screening, we applied the Pharmacophore Search as a screening protocol to identify active compounds that have good matching with query features of the generated pharmacophore model (Zhou et al., 2019a). Hit molecules can be ranked by their RMSDx values which indicate how well the matching ligand annotation points of the chemical structures were mapped onto the query features of the model. A low RMSDx value indicates a good matching of the ligand annotation points with query features of the model.

### 2.4 Molecular docking experiments

In this study, we used the 3CL^pro^ structure from SARS-CoV-2 with ML300 (PDB code: 7LME) from the pharmacophore study (Han et al., 2022). It has high resolution and relatively complete structure, so it is used as the docking model. By using default settings, the hit compounds obtained were docked into the 3CL^pro^ active site containing some amino acid residues (such as Phe140, Leu141, Met165, Met49, Thr25, and Thr24) through the Triangle Matcher Docking protocol of MOE (Zhou et al., 2019a; Zhou et al., 2019b). Amber10:EHT forcefield was assigned to the system. The dG docking scoring function are selected to rank the compounds.

### 2.5 In silico ADME studies

ADMETlab web server (https://admetmesh.scbdd.com/) was used to predict the ADME properties of selected hits (Tian et al., 2022). The molecular weight (mol_MW), number of hydrogen bond acceptors (nHA), number of hydrogen bond donors (nHD), log of the octanol/water partition coefficient (logP), and log of the aqueous solubility (LogS) were evaluated.

### 2.6 Molecular dynamics simulations

According to a previously reported method (Tian et al., 2022), protein-hit complexes were investigated by molecular dynamics simulation using Groningen machine for chemical simulations software (GROMACS).

### 2.7 Microscale thermophoresis (MST) assay

The purified 3CL^pro^ protein was purchased from Abcam (Cambridge, MA, United States). According to a previously reported method (Hang et al., 2019), the binding affinity of the compounds with SARS-CoV-2 3CL^pro^ was detected by MST assay. Briefly, purified SARS-CoV-2 3CL^pro^ was labelled with the Monolith NT Protein Labelling Kit RED (NanoTemper Technologies). Serially diluted compounds, with concentrations of 0.76 nM–25 μM, were mixed with 100 nM labelled SARS-CoV-2 3CL^pro^ at room temperature and loaded into Monolith standard-treated capillaries. The fluorescent signal was detected by Monolith NT.115 (NanoTemper, Munich, Germany). The *K*_d_ value was calculated by fitting a standard binding curve. Experiment was performed in triplicate.

### 2.8 Inhibitory effects on SARS-CoV-2-3CL^pro^ activity

According to a previously reported method (Han et al., 2022), 200 nM of SARS-CoV-2 3CL^pro^ were incubated with different concentrations of compounds (0, 0.19, 0.76, 3.05, 12.21, 48.83, 195.31, 781.25, 3125, 12,500, and 50,000 nM) for 20 min. The reaction was initiated by adding fluorophore-quencher peptide substrate HiyteFluor-488ESATLQSGLRKAK-(QXL)-NH_2_, followed by 30 min incubation at 25°C. Fluorescence intensity was measured on a microplate reader (λex = 485 nm; λem = 528 nm). Experiment was performed in triplicate.

## 3 Results and discussion

### 3.1 Pharmacophore model generation and validation

**FIGURE 1:**
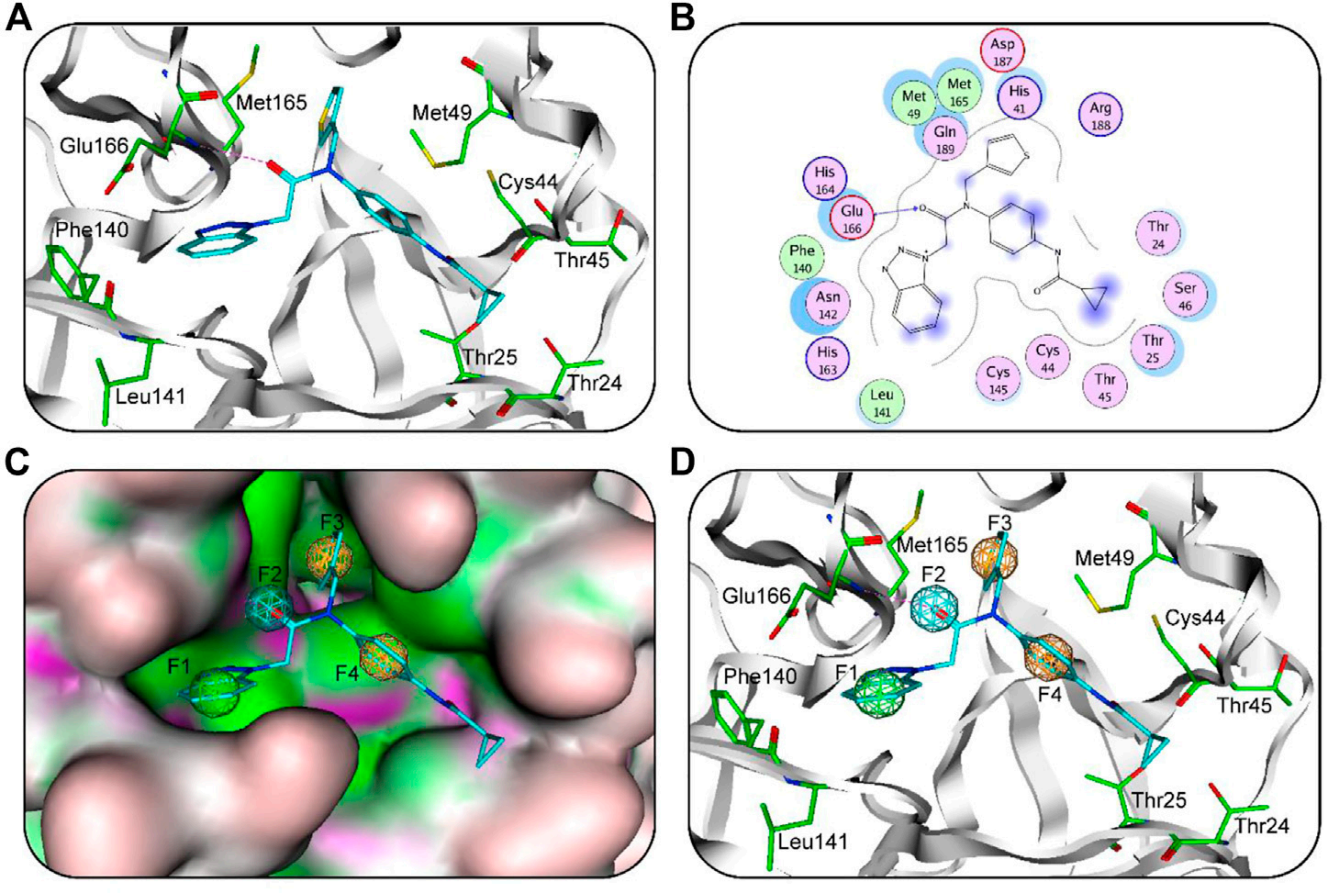
**(A)** 3D binding mode of ML300 and active-site residues of 3CL^pro^. Cyan stick represent ML300 and green color represents residues; tube form represents the protein backbone; Red dotted line represents the hydrogen-bonding interactions. **(B)** 2D binding mode of ML300 and active-site residues of 3CLpro. Blue dotted line indicates hydrogen-bond interaction; Pink represents polar amino acid residues; green represents greasy amino acid residues. **(C)** The generated 3CLpro-pharmacophore model. **(D)** The 3CLpro-model mapped onto ML300. Green, cyan, and orange represent F1 hydrophobic feature, F2 hydrogen-bond acceptor feature and two aromatic features (F3 and F4), respectively.

Recently, researchers have reported the structural complex of SARS-CoV-2 3CL^pro^ with ML300 (PDB code: 7LME) (Han et al., 2022). Therefore, we used this structure analyze the chemical features of 3CL^pro^-ML300 interaction (Figure 1A). Based on their interaction between 3CL^pro^ and ML300 (Figure 1B), the complex structure was used to generate a 3CL^pro^-pharmacophore model (Figure 1C). This model consists of four features: F1 hydrophobic feature, F2 hydrogen-bond acceptor feature, F3 aromatic feature, and F4 aromatic feature. The F1 feature mapped with its aromatic ring of ML300 described hydrophobic interactions with residues Phe140 and Leu141 in the 3CL^pro^-active site (Figure 1D). The F2 feature mapped with the oxygen atom of ML300, formed an important hydrogen-bond interaction between ML300 and Glu166, while the aromatic rings of ML300 mapped on the aromatic features are involved in interactions with hydrophobic residues Met165 and Met49. The results indicates that the 3CL^pro^-model features could effectively map the interactions between ligands and 3CL^pro^-active residues.

### 3.2 Pharmacophore model Validation

The quality of 3CL^pro^-model was validated using GH scoring method (Tian et al., 2022). The GH analysis were done by computing statistical parameters such as the enrichment factor (*E*) and goodness of hit score (*GH*). The 3CL^pro^-model was successful in retrieving 90% of active compounds from the decoys set (Table 1). Moreover, an enrichment factor of 89 and a GH score of 0.84 indicated good quality of the model. The result reveals that the model can distinguish active inhibitors of 3CL^pro^ from the decoy set.

**TABLE 1:** Statistical parameter from pharmacophore-based virtual screening using GH scoring method.

Serial no	Parameter	Pharmacophore model
1	Total molecules in database(D)	1090
2	Total number of actives in database(A)	10
3	Total hits(Ht)	11
4	Achive hits(Ha)	9
5	%Yield of actives [(Ha/Ht)*100]	82%
6	%Ratio of actives [(Ha/A)*100]	90%
7	Enrichment factor (E)[(Ha*D)/(Ht*A)]	89
8	False negatives[A-Ha]	1
9	False positives[Ht-Ha]	2
10	Goodness of hit score(GH)	0.84

### 3.3 Virtual screening

**FIGURE 2:**
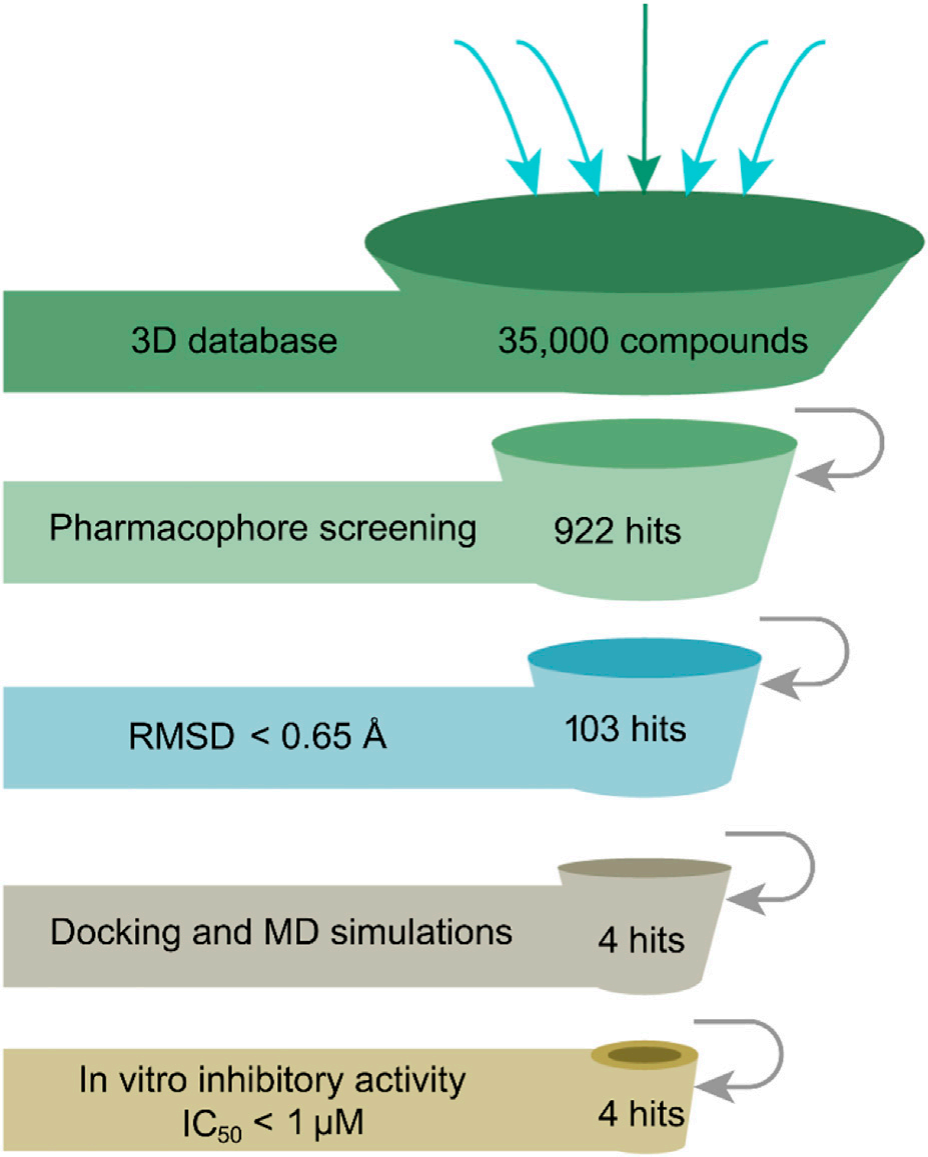
Workflow of structure-based virtual searching approach.

**FIGURE 3:**
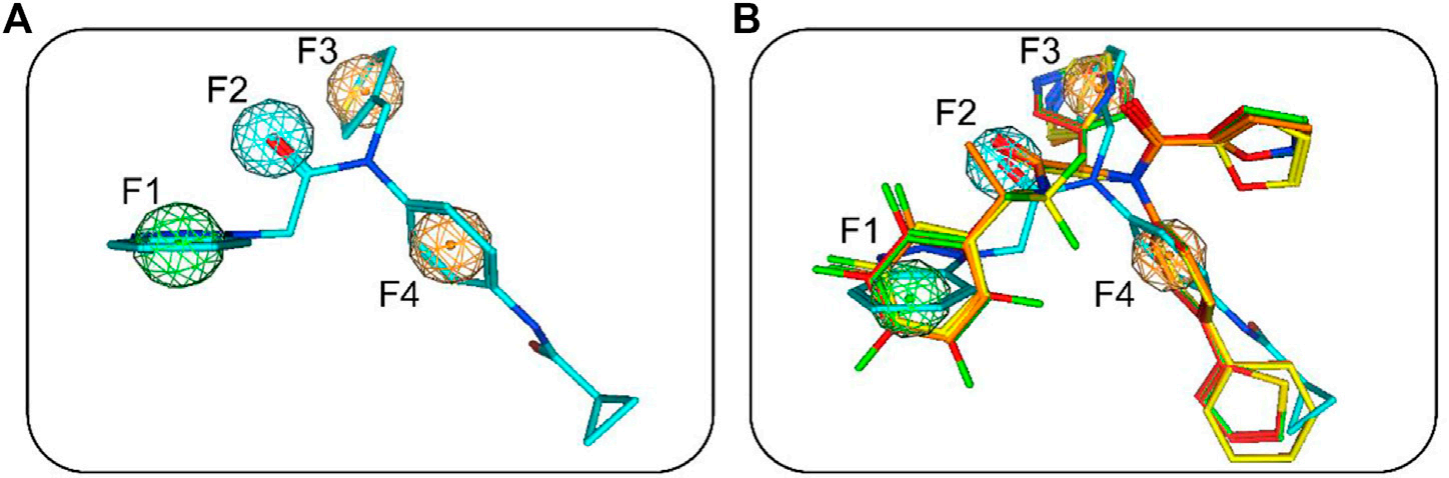
**(A)** SARS-CoV-2-3CL^pro^-model mapping with ML300. **(B)** Superimposition of ML300 and hits one to four with 3CL^pro^-model. Green, cyan, and orange represent F1 hydrophobic feature, F2 hydrogen-bond acceptor feature and two aromatic features (F3 and F4), respectively.

The flowchart in Figure 2 is a detailed schematic diagram of our virtual screening process in this study. The validated SARS-CoV-2-3CL^pro^-model is applied as a 3D search query to virtually screen a 35,000-compound database. The obtained 922 compounds that could be mapped onto 3CL^pro^-model model were successfully retrieved, including 103 compounds with RMSDx values lower than 0.65 Å (the lower the value, the better the pharmacophore mapping). Subsequently, the retrieved 103 compounds were docked into the 3CL^pro^-active site to reduce the false positive molecules. The 3CL^pro^ inhibitor ML300 with a docking score of -10.90 kcal/mol was used as a positive control. Finally, the top four hits (hits 1–4) with lower docking scores (lower than -10.90 kcal/mol) were selected (Table 2). The analysis of the model matching results of four hit compounds showed that the oxygen atoms of four hit compounds matched the F2 hydrogen-bond acceptor feature of 3CL^pro^-model while their two aromatic rings were mapped with the F3 and F4 aromatic features, respectively (Figure 3). Moreover, the hydrophobic groups of four hits matched the features of F1 hydrophobic feature. The superimposition results indicated that the four structurally similar hits match the 3CL^pro^-model very well. The structural and chemical similarities of four hits suggested good pharmacophore mapping and docking scores. Furthermore, additional characterization of hits one to four such as the molecular weight (mol_MW), number of hydrogen bond acceptors (nHA), number of hydrogen bond donors (nHD), log of the octanol/water partition coefficient (logP), and log of the aqueous solubility (LogS) was evaluated and their parameter values are in the optimal range (Supplementary Table S2), suggesting their druggable and pharmacokinetics properties. Finally, four candidate hits (hits 1–4) were used as candidate molecules for performing molecular dynamics simulations.

**TABLE 2:** Virtual screening parameters and biological activity data of four hits.

	Chemical structure	RMSDx [Å]	Docking score [kcal/mol]b)	IC50 [µM]
Hit 1	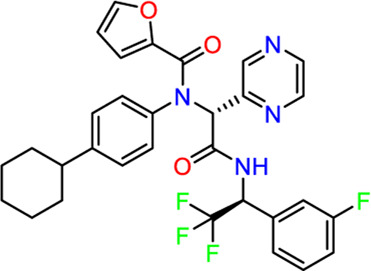	0.6033	-11.26	0.017 ± 0.003
Hit 2	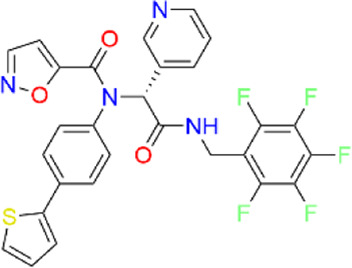	0.6147	-10.93	0.83 ± 0.11
Hit 3	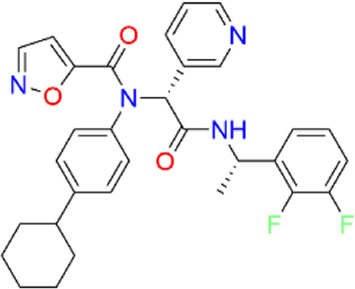	0.6086	-11.04	0.09 ± 0.02
Hit 4	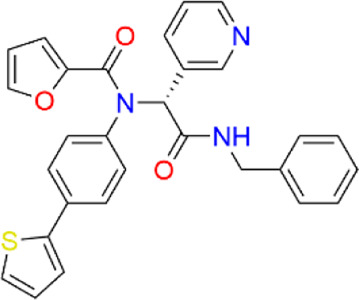	0.6112	-10.98	0.75 ± 0.04
ML300	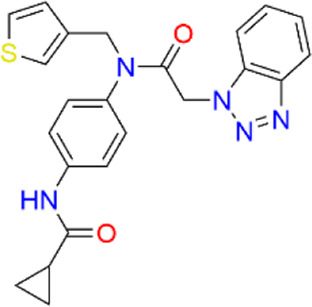		-10.90	4.01 ± 0.66

### 3.4 Molecular dynamics (MD) simulations

**FIGURE 4:**
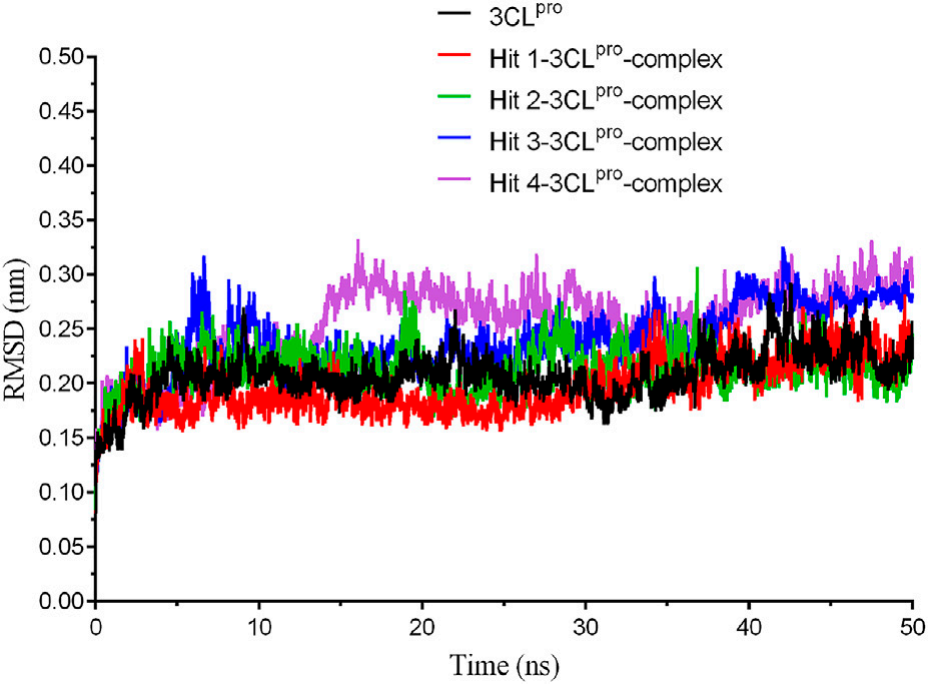
The root-mean-square deviation (RMSD) trajectories of 3CL^pro^ protein and each hit-3CL^pro^ complex during 50 ns simulations.

**FIGURE 5:**
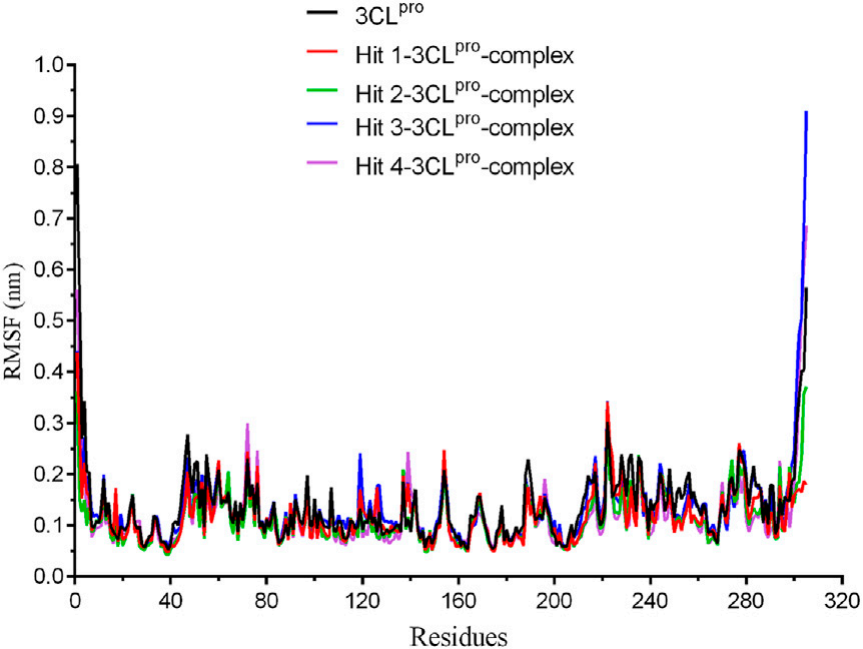
The root-mean-square fluctuation (RMSF) of amino acid residues from 3CL^pro^ protein and each hit-3CL^pro^ complex during 50 ns simulations.

To evaluate the binding stability of each 3CL^pro^-hit complex from the docking study, we conducted 50 ns MD simulation to analyze the parameters such as root mean square deviation (RMSD) and root mean square fluctuation (RMSF). As shown in Figure 4, the movement of 3CL^pro^ protein and each hit-3CL^pro^ complex was monitored. We observed that 3CL^pro^ protein and each 3CL^pro^-hit complex can exhibit stable internal motion throughout the simulation process. The RMSF was also calculated that provides knowledge on the flexibility of the protein residues. Figure 5 shows that compared with that of 3CL^pro^ protein alone, the RMSF fluctuation values of the active residues (Glu166, Phe140, Leu141, Met165, Met49, Thr25, and Thr24) from all hit-3CL^pro^ complexes were relatively small. In addition, we also monitor the movement of each ligand in the four complexes. As depicted in Supplementary Figure S1, the RMSD value for each individual hit (from each 3CL^pro^-hit complex during MD simulation) is below 0.33 nm, indicating its stability at the binding site. Overall, these data show that four hits can stably bind to the active site of 3CL^pro^.

### 3.5 Inhibitory effects on SARS-CoV-2-3CL^pro^ activity

To characterize hits one to four targeting 3CL^pro^ *in vitro*, we used the MST assay to measure the binding affinity of hits one to four to 3CL^pro^. The previously reported 3CL^pro^ inhibitor ML300 was used as a positive control. MST assay showed that the K_d_ values of hits one to four to 3CL^pro^ were ranged from 0.012 μM to 0.27 μM (Supplementary Figure S2). We also evaluated the inhibition effect of hits one to four on 3CL^pro^ by enzyme inhibition assay. As shown in Table 2 and Supplementary Figure S3, all the four hits inhibited SARS-CoV-2-3CL^pro^ activity, with IC_50_ values ranging from 0.017 to 0.83 μM. In particular, hit one is a promising 3CL^pro^ inhibitor. Its inhibition activity (IC_50_ = 0.017 ± 0.003 µM) was about 236 times stronger than that of ML300 (IC_50_ = 4.01 ± 0.66 µM). Through the search analysis of PubChem and SciFinder, it was found that hits one to four with the inhibition activity of 3CL^pro^ were reported for the first time, indicating that they are novel SARS-CoV-2-3CL^pro^ inhibitors. These experimental data demonstrate that our virtual screening protocol is very reliable in identifying novel and effective 3CL^pro^ inhibitors.

### 3.6 Analysis of the interaction mode

**FIGURE 6:**
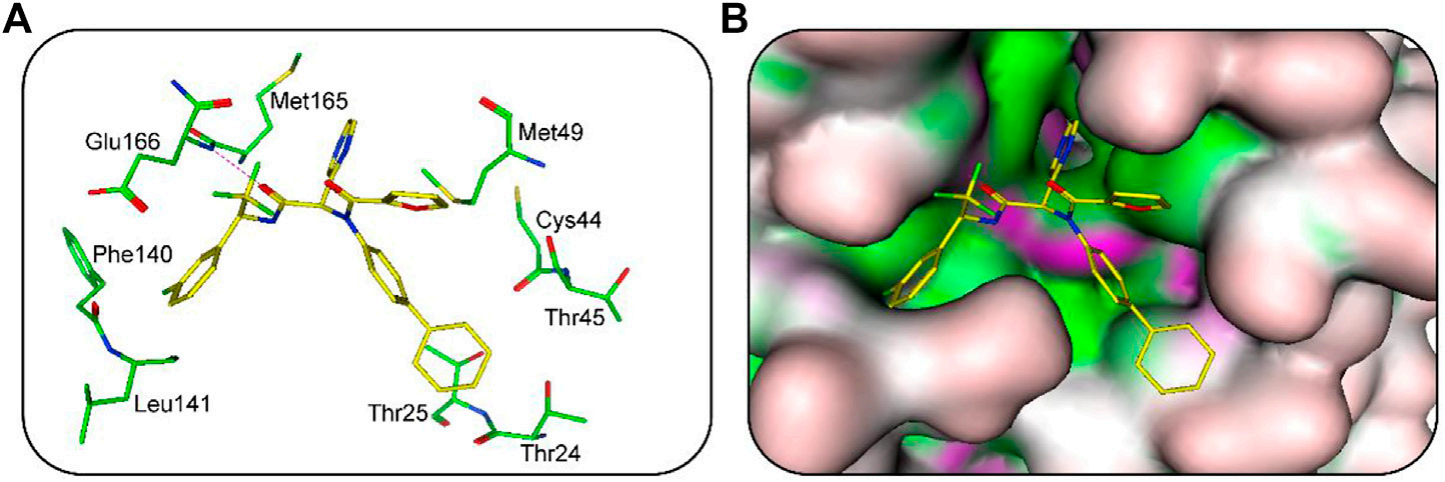
**(A)** Predicted binding mode of hit 1 (yellow, sticks) and the 3CL^pro^ active-site residues (green, sticks). The hydrogen bonds are illustrated as red dotted lines. **(B)** Binding mode of 3CL^pro^-hit-1 complex.

The binding modes of hit 1 with SARS-CoV-2-3CL^pro^ were further analyzed. The oxygen atom of hit one formed a hydrogen-bond interaction with Glu166 and hydrophobic interactions with key residues including Phe140, Leu141, Met165, Met49, Thr25, and Thr24 (Figure 6A). As shown in Figure 6B, it can be observed that the 3CL^pro^-active site has multiple hydrophobic sites, and the active compound targeting SARS-CoV-2-3CL^pro^ should occupy each site at the same time. Our molecular docking results indicated that hit one showed excellent geometric matching with SARS-CoV-2-3CL^pro^. In addition, we also measured the inter-atomic distance profile of the important interacting atom pairs (hydrogen bond: for details see Supplementary Table S3) of the 3CL^pro^ and hits during the MD simulation. The result indicates that the oxygen atoms of all hits can form hydrogen-bond interactions with Glu166. In order to further evaluate the stability of the 3CL^pro^ in complex with hits one to four, we also used DSSP algorithm to monitor the secondary structure changes of each 3CL^pro^-hit complex during MD simulation (Supplementary Figure S4). The result suggests that the changes of structural elements (such as α- Helix and β-Sheet content) were not observed, indicating the stability of each hit-complex system. As shown in Supplementary Figure S5, it can be clearly observed that the 3CL^pro^ complexes superpose well with the 3CL^pro^ alone with a Cα-RMSD < 2.5 Å, indicating that all systems retained the structural integrity and maintained the same fold with minor changes of the loop region in the 3CL^pro^ of SARS-Cov-2.

## 4 Conclusion

With the fourth wave of COVID-19 sweeping the world, the development of COVID-19 therapeutics is still a major challenge. The 3CL^pro^ plays a vital role in viral replication and has hence been considered as a potent drug target for SARS-CoV-2 infection. However, the development of highly effective 3CL^pro^ inhibitors has made limited progress. Here, we proposed a combined screening strategy of structure-based virtual screening including model mapping, molecular docking and MD simulation. Through this virtual screening approach, four potential hit compounds were identified. The bioassay showed that all hits effectively inhibited 3CL^pro^ with IC_50_ values less than 1 μM. Particularly, hit one showed highly potent 3CL^pro^ inhibition activity at nanomolar concentration. These promising results illustrated that hit one could be a candidate prototype worth exploring further for the treatment of COVID-19.

## Data Availability

The original contributions presented in the study are included in the article/Supplementary Material, further inquiries can be directed to the corresponding authors.

## References

[B1] Abdelnabi R. Foo C. S. Jochmans D. Vangeel L. De Jonghe S. Augustijns P. (2022). The oral protease inhibitor (PF-07321332) protects Syrian hamsters against infection with SARS-CoV-2 variants of concern. Nat. Commun. 13, 719. 10.1038/s41467-022-28354-0 35169114 PMC8847371

[B2] Adegbola P. I. Semire B. Fadahunsi O. S. Adegoke A. E. (2021). Molecular docking and ADMET studies of Allium cepa, Azadirachta indica and Xylopia aethiopica isolates as potential anti-viral drugs for Covid-19. Virusdisease 32, 85–97. 10.1007/s13337-021-00682-7 33869672 PMC8036013

[B3] Boras B. Jones R. M. Anson B. J. Arenson D. Aschenbrenner L. Bakowski M. A. (2021). Preclinical characterization of an intravenous coronavirus 3CL protease inhibitor for the potential treatment of COVID19. Nat. Commun. 12, 6055. 10.1038/s41467-021-26239-2 34663813 PMC8523698

[B4] Chakraborty C. Bhattacharya M. Sharma A. R. (2022). Emerging mutations in the SARS-CoV-2 variants and their role in antibody escape to small molecule-based therapeutic resistance. Curr. Opin. Pharmacol. 62, 64–73. 10.1016/j.coph.2021.11.006 34920267 PMC8606259

[B5] Ferreira J. C. Fadl S. Ilter M. Pekel H. Rezgui R. Sensoy O. (2021). Dimethyl sulfoxide reduces the stability but enhances catalytic activity of the main SARS-CoV-2 protease 3CLpro. Faseb J. 35, e21774. 10.1096/fj.202100994 34324734 PMC8441638

[B6] Gao Q. Yang L. Zhu Y. (2010). Pharmacophore based drug design approach as a practical process in drug discovery. Curr. Comput. Aided. Drug Des. 6, 37–49. 10.2174/157340910790980151 20370694

[B7] Guan W. J. Ni Z. Y. Hu Y. Liang W. H. Ou C. Q. He J. X. (2020). Clinical characteristics of coronavirus disease 2019 in China. N. Engl. J. Med. 382, 1708–1720. 10.1056/NEJMoa2002032 32109013 PMC7092819

[B8] Han S. H. Goins C. M. Arya T. Shin W. J. Maw J. Hooper A. (2022). Structure-based optimization of ml300-derived, noncovalent inhibitors targeting the severe acute respiratory syndrome coronavirus 3CL protease (SARS-CoV-2-3CL(pro)). J. Med. Chem. 65, 2880–2904. 10.1021/acs.jmedchem.1c00598 34347470 PMC8353992

[B9] Hang S. Paik D. Yao L. Kim E. Trinath J. Lu J. (2019). Bile acid metabolites control TH17 and Treg cell differentiation. Nature 576, 143–148. 10.1038/s41586-019-1785-z 31776512 PMC6949019

[B10] Hu Q. Xiong Y. Zhu G. H. Zhang Y. N. Zhang Y. W. Huang P. (2022). The SARS-CoV-2 main protease (M(pro)): Structure, function, and emerging therapies for COVID-19. MedComm 3, e151. 10.1002/mco2.151 35845352 PMC9283855

[B11] Jin Z. Du X. Xu Y. Deng Y. Liu M. Zhao Y. (2020). Structure of M(pro) from SARS-CoV-2 and discovery of its inhibitors. Nature 582, 289–293. 10.1038/s41586-020-2223-y 32272481

[B12] Kitchen D. B. Decornez H. Furr J. R. Bajorath J. (2004). Docking and scoring in virtual screening for drug discovery: Methods and applications. Nat. Rev. Drug Discov. 3, 935–949. 10.1038/nrd1549 15520816

[B13] Koutsakos M. Kedzierska K. (2020). A race to determine what drives COVID-19 severity. Nature 583, 366–368. 10.1038/d41586-020-01915-3 32661414

[B14] Maga G. Falchi F. Garbelli A. Belfiore A. Witvrouw M. Manetti F. (2008). Pharmacophore modeling and molecular docking led to the discovery of inhibitors of human immunodeficiency virus-1 replication targeting the human cellular aspartic acid-glutamic acid-alanine-aspartic acid box polypeptide 3. J. Med. Chem. 51, 6635–6638. 10.1021/jm8008844 18834110

[B15] Mysinger M. M. Carchia M. Irwin J. J. Shoichet B. K. (2012). Directory of useful decoys, enhanced (DUD-E): Better ligands and decoys for better benchmarking. J. Med. Chem. 55, 6582–6594. 10.1021/jm300687e 22716043 PMC3405771

[B16] Narayanan A. Narwal M. Majowicz S. A. Varricchio C. Toner S. A. Ballatore C. (2022). Identification of SARS-CoV-2 inhibitors targeting Mpro and PLpro using in-cell-protease assay. Commun. Biol. 5, 169. 10.1038/s42003-022-03090-9 35217718 PMC8881501

[B17] Nassau D. E. Best J. C. Kresch E. Gonzalez D. C. Khodamoradi K. Ramasamy R. (2022). Impact of the SARS-CoV-2 virus on male reproductive health. BJU Int. 129, 143–150. 10.1111/bju.15573 34402155 PMC8444635

[B18] Rockett R. Basile K. Maddocks S. Fong W. Agius J. E. Johnson-Mackinnon J. (2022). Resistance mutations in SARS-CoV-2 delta variant after sotrovimab use. N. Engl. J. Med. 386, 1477–1479. 10.1056/NEJMc2120219 35263515 PMC8929376

[B19] Stevens L. J. Pruijssers A. J. Lee H. W. Gordon C. J. Tchesnokov E. P. Gribble J. (2022). Mutations in the SARS-CoV-2 RNA-dependent RNA polymerase confer resistance to remdesivir by distinct mechanisms. Sci. Transl. Med. 14, eabo0718. 10.1126/scitranslmed.abo0718 35482820 PMC9097878

[B20] Tao K. Tzou P. L. Nouhin J. Gupta R. K. de Oliveira T. Kosakovsky Pond S. L. (2021). The biological and clinical significance of emerging SARS-CoV-2 variants. Nat. Rev. Genet. 22, 757–773. 10.1038/s41576-021-00408-x 34535792 PMC8447121

[B21] Tian X. Zhao Q. Chen X. Peng Z. Tan X. Wang Q. (2022). Discovery of novel and highly potent inhibitors of SARS CoV-2 papain-like protease through structure-based pharmacophore modeling, virtual screening, molecular docking, molecular dynamics simulations, and biological evaluation. Front. Pharmacol. 13, 817715. 10.3389/fphar.2022.817715 35264955 PMC8899470

[B22] Tyndall J. D. A. S (2022). S-217622, a 3CL protease inhibitor and clinical candidate for SARS-CoV-2. J. Med. Chem. 65, 6496–6498. 10.1021/acs.jmedchem.2c00624 35507419

[B23] Vuong W. Khan M. B. Fischer C. Arutyunova E. Lamer T. Shields J. (2020). Feline coronavirus drug inhibits the main protease of SARS-CoV-2 and blocks virus replication. Nat. Commun. 11, 4282. 10.1038/s41467-020-18096-2 32855413 PMC7453019

[B24] Wang J. Kang X. Kuntz I. D. Kollman P. A. (2005). Hierarchical database screenings for HIV-1 reverse transcriptase using a pharmacophore model, rigid docking, solvation docking, and MM-PB/SA. J. Med. Chem. 48, 2432–2444. 10.1021/jm049606e 15801834

[B25] Wieder M. Garon A. Perricone U. Boresch S. Seidel T. Almerico A. M. (2017). Common hits approach: Combining pharmacophore modeling and molecular dynamics simulations. J. Chem. Inf. Model. 57, 365–385. 10.1021/acs.jcim.6b00674 28072524

[B26] Xia B. Kang X. (2011). Activation and maturation of SARS-CoV main protease. Protein Cell 2, 282–290. 10.1007/s13238-011-1034-1 21533772 PMC4875205

[B27] Zehra Z. Luthra M. Siddiqui S. M. Shamsi A. Gaur N. A. Islam A. (2020). Corona virus versus existence of human on the Earth: A computational and biophysical approach. Int. J. Biol. Macromol. 161, 271–281. 10.1016/j.ijbiomac.2020.06.007 32512089 PMC7273167

[B28] Zheng L. Ren R. Sun X. Zou Y. Shi Y. Di B. (2021). Discovery of a dual tubulin and poly(ADP-ribose) polymerase-1 inhibitor by structure-based pharmacophore modeling, virtual screening, molecular docking, and biological evaluation. J. Med. Chem. 64, 15702–15715. 10.1021/acs.jmedchem.1c00932 34670362

[B29] Zhou Y. Chen Y. Tan Y. Hu R. Niu M. M. (2021). An NRP1/MDM2-targeted D-peptide supramolecular nanomedicine for high-efficacy and low-toxic liver cancer therapy. Adv. Healthc. Mat. 10, e2002197. 10.1002/adhm.202002197 33690977

[B30] Zhou Y. Di B. Niu M. M. (2019). Structure-based pharmacophore design and virtual screening for novel tubulin inhibitors with potential anticancer activity. Molecules 24, E3181. 10.3390/molecules24173181 PMC6749218 31480625

[B31] Zhou Y. Tang S. Chen T. Niu M. M. (2019). Structure-based pharmacophore modeling, virtual screening, molecular docking and biological evaluation for identification of potential poly (ADP-Ribose) polymerase-1 (PARP-1) inhibitors. Molecules 24, E4258. 10.3390/molecules24234258 PMC6930522 31766720

[B32] Zhou Y. Zou Y. Yang M. Mei S. Liu X. Han H. (2022). Highly potent, selective, biostable, and cell-permeable cyclic d-peptide for dual-targeting therapy of lung cancer. J. Am. Chem. Soc. 144, 7117–7128. 10.1021/jacs.1c12075 35417174

