# Supplementary material for: Identification of highly effective inhibitors against SARS-CoV-2 main protease: From virtual screening to in vitro study
Source: Front Pharmacol. 2022 Nov 18;13:1036208. doi: 10.3389/fphar.2022.1036208 (PMC9715617; doi:10.3389/fphar.2022.1036208)
Supplement: Supplementary file 1 [file DataSheet1.docx]

Supplementary Material

Identification of Highly Effective Inhibitors Against SARS-CoV-2 Main Protease: From Virtual Screening to In Vitro Study

Hu Wang,^+[a]^ Jun Wen,^+[a]^ Yang Yang,^[b]^ Hailin Liu,^[a]^ Song Wang,^[a]^ Xiaoli Ding,^[a]^ Chunqiao Zhou,*^[a]^ Xuelin Zhang,*^[a]^

[a] H. Wang, J. Wen, H. Liu, S. Wang, X. Ding, C. Zhou, and X. Zhang
Department of Pharmacy
The First People’s Hospital of Chongqing Liang Jiang New Area
Chongqing 401121, China
E-mail: zhangxuelin719@163.com; zcqiao163@163.com

[b] Dr. Y. Yang
Department of Pharmacology
Chongqing Health Center for Women and Children
Chongqing 401147, China

[+] *These authors contributed equally to this work.*

[*] *Corresponding author.*

**Table S1.** The drugs targeting SARS-CoV-2-3CL^pro^

| **Name** | **Company** | **Delivery** | **States** |
| --- | --- | --- | --- |
| PF-07321332 (Paxlovir) | Pfizer | Oral | Approved |
| S-217622 | Shionogi | Oral | Phase III |
| PF-07304814 | Pfizer | IV | Phase I |
| EDP-235 | Enanta | Oral | Phase I |
| JTT-705 RG-1658(Dalcetrapib) | DalCor Pharmaceuticals | – | Phase II |
| FB2001/11a (DC402234) | Frontier | IV | Phase I |
| SIM0417 (SSD8432) | Simcere | Oral | Phase II |
| PBI-0451 | Pardes | Oral | Phase I |

**Table S2.** The drug-like filter properties of identified hits in virtual screen.

| **Lipinski’s filter** | **Hit 1** | **Hit 2** | **Hit 3** | | | **Hit 4** |  |
| --- | --- | --- | --- | --- | --- | --- | --- |
| mol_MW | 580.21 | 584.09 | | 544.23 | 493.15 | |  |
| nHA | 7.00 | 7.00 | | 7.00 | 6.00 | |  |
| nHD | 1.00 | 1.00 | | 1.00 | 1.00 | |  |
| LogS | -4.75 | -5.20 | | -4.67 | -4.57 | |  |
| LogP | 5.84 | 4.55 | | 5.46 | 4.47 | |  |

mol_MW: molecular weight, optimal:100~600; nHA: number of hydrogen bond acceptors, optimal: 0~10; nHD: number of hydrogen bond donors, optimal: 0~5; LogS: Log of the aqueous solubility, optimal: -6~0.5; LogP: log of the octanol/water partition coefficient, optimal: 0~6.

**Table S3.** Hydrogen bond and its occupancy formed in the binding of each hit and 3CL^pro^ during the MD simulation.

| **Acceptor** | **Hydrogen** | **Donor** | **Occupancy%** |
| --- | --- | --- | --- |
| **Hit 1** | | | |
| Hit 1@O2 | Glu166@H | Glu166@N | 74.4 |
| **Hit 2** | | | |
| Hit 2@O2 | Glu166@H | Glu166@N | 77.1 |
| **Hit 3** | | | |
| Hit 3@O2 | Glu166@H | Glu166@N | 93.1 |
| **Hit 4** | | | |
| Hit 4@O2 | Glu166@H | Glu166 @N | 67.0 |

**

**

**Figure S1.** The RMSD trajectories of each individual hit during 50 ns simulations.


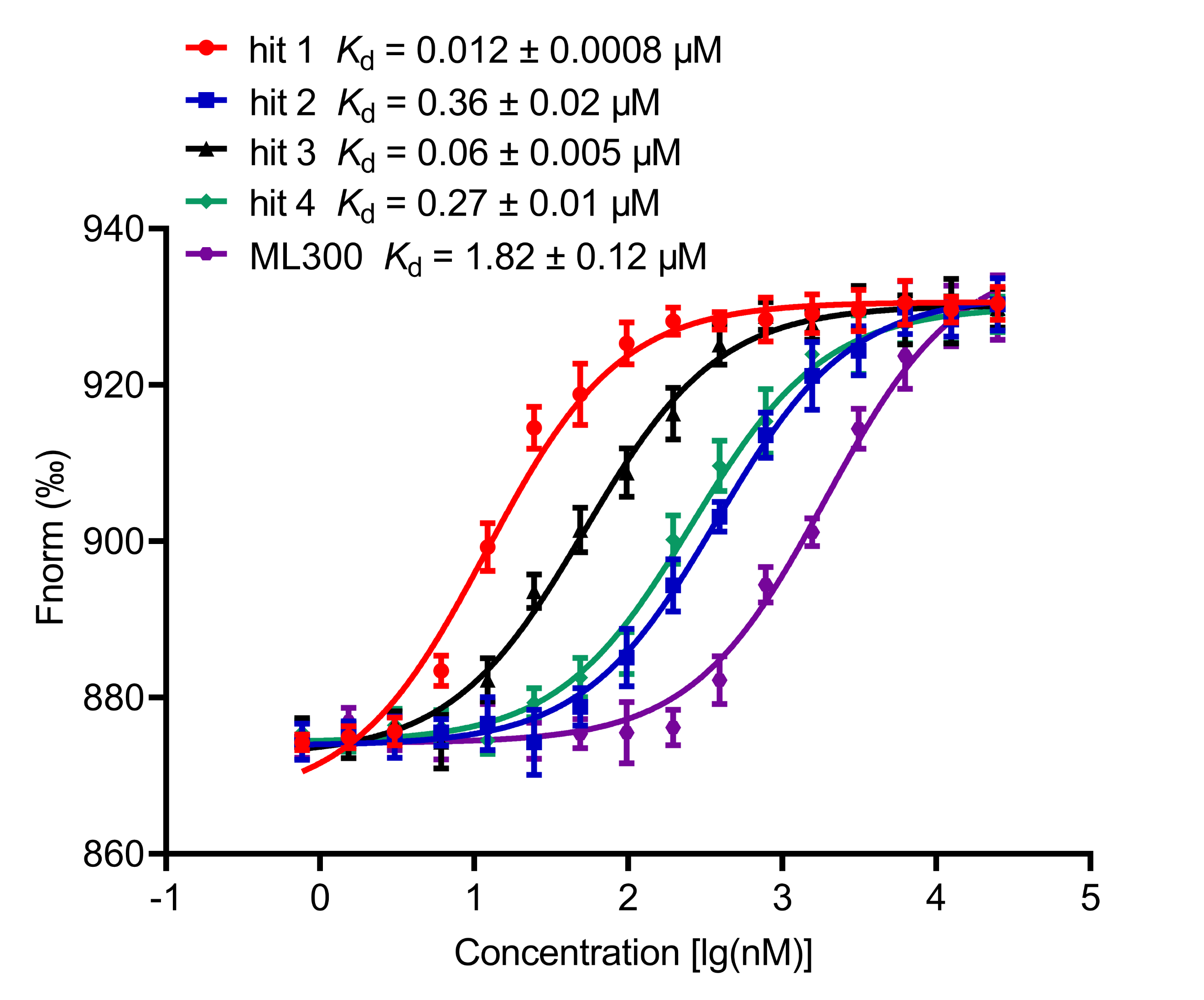


**Figure S2.** Binding affinity of hits 1-4 and ML300 to 3CL^pro^.


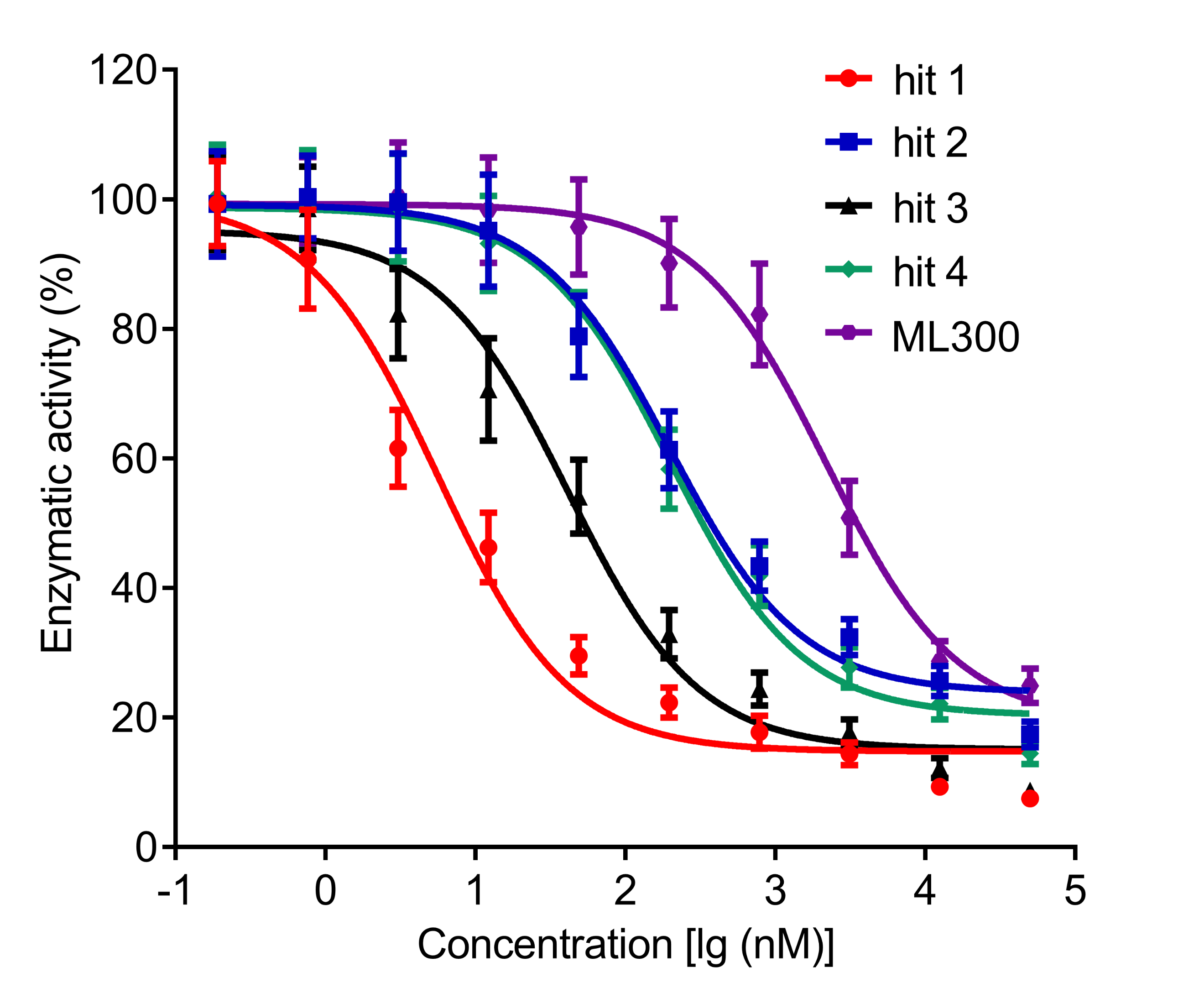


**Figure S3.** 3CL^pro^ concentration−response curves for hits 1-4 and ML300.

**
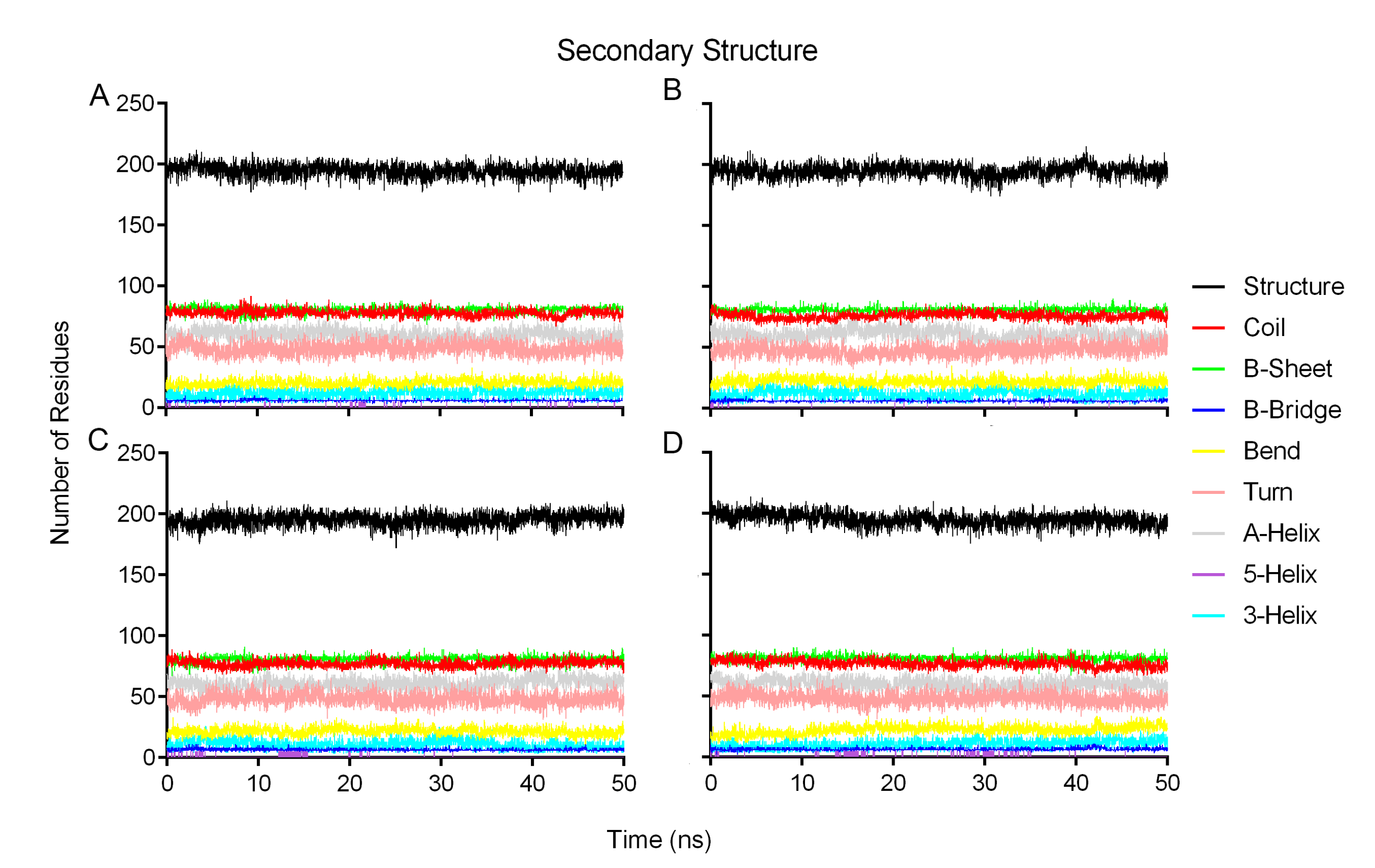
**

**Figure S4.** Dynamics of evolution of secondary structure elements of the 3CL^pro^ complex with hit 1 (A), hit 2 (B), hit 3 (C), and hit4 (D) during the MD simulations.


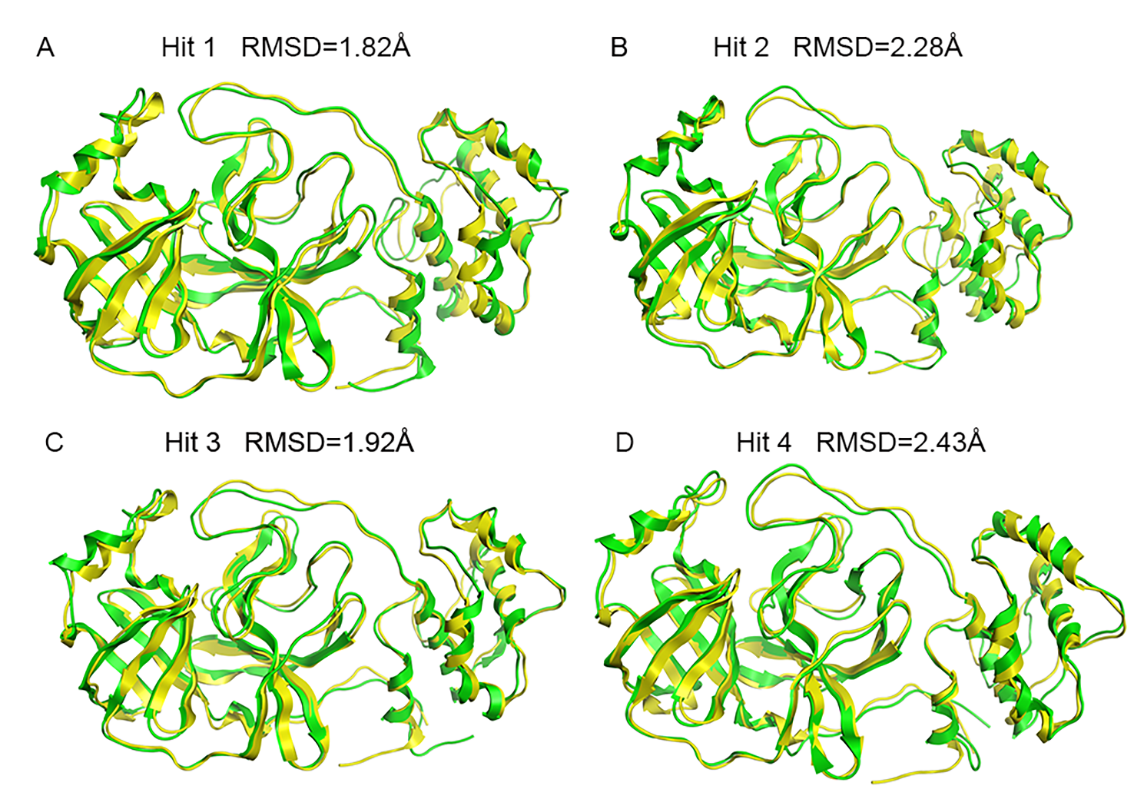


**Figure S5.** Structural superimposition of the top ranked cluster representative of 3CL^pro^-hit complexes (shown in green) obtained from MD simulation as compared to the 3CL^pro^ alone (Yellow) without any ligand. (A) Secondary structural changes of 3CL^pro^ protein upon binding to hit 1. (B) Secondary structural changes of 3CL^pro^ protein upon binding to hit 2. (C) Secondary structural changes of 3CL^pro^ protein upon binding to hit 3. (D) Secondary structural changes of 3CL^pro^ protein upon binding to hit 4.
